# A New Role of the Complement System: C3 Provides Protection in a Mouse Model of Lung Infection with Intracellular *Chlamydia psittaci*

**DOI:** 10.1371/journal.pone.0050327

**Published:** 2012-11-26

**Authors:** Jenny Bode, Pavel Dutow, Kirsten Sommer, Katrin Janik, Silke Glage, Burkhard Tümmler, Antje Munder, Robert Laudeley, Konrad W. Sachse, Andreas Klos

**Affiliations:** 1 Institute of Medical Microbiology and Hospital Epidemiology, Hannover Medical School (MHH), Hannover, Germany; 2 Institute for Laboratory Animal Science, Hannover Medical School (MHH), Hannover, Germany; 3 Clinical Research Group, Pediatric Pulmonology and Neo­nato­logy, Hannover Medical School (MHH), Hannover, Germany; 4 Friedrich-Loeffler-Institut (Federal Research Institute for Animal Health), Jena, Germany; University of Iowa Carver College of Medicine, United States of America

## Abstract

The complement system modulates the intensity of innate and specific immunity. While it protects against infections by extracellular bacteria its role in infection with obligate intracellular bacteria, such as the avian and human pathogen *Chlamydia (C.) psittaci*, is still unknown. In the present study, knockout mice lacking C3 and thus all main complement effector functions were intranasally infected with *C. psittaci* strain DC15. Clinical parameters, lung histology, and cytokine levels were determined. A subset of infections was additionally performed with mice lacking C5 or C5a receptors. Complement activation occurred before symptoms of pneumonia appeared. Mice lacking C3 were ∼100 times more susceptible to the intracellular bacteria compared to wild-type mice, with all C3^−/−^ mice succumbing to infection after day 9. At a low infective dose, C3^−/−^ mice became severely ill after an even longer delay, the kinetics suggesting a so far unknown link of complement to the adaptive, protective immune response against chlamydiae. The lethal phenotype of C3^−/−^ mice is not based on differences in the anti-chlamydial IgG response (which is slightly delayed) as demonstrated by serum transfer experiments. In addition, during the first week of infection, the absence of C3 was associated with partial protection characterized by reduced weight loss, better clinical score and lower bacterial burden, which might be explained by a different mechanism. Lack of complement functions downstream of C5 had little effect. This study demonstrates for the first time a strong and complex influence of complement effector functions, downstream of C3 and upstream of C5, on the outcome of an infection with intracellular bacteria, such as *C. psittaci*.

## Introduction

*Chlamydia* (*C.*) *psittaci* is an important pathogen in birds and other animals. Following transmission from avian sources, it can cause psittacosis in humans, a life-threatening atypical pneumonia with systemic spread [1]. Infections of cattle, sheep, horses and other domestic animals with this agent can result in abortion, respiratory disorders, enteritis and arthritis [2]. Even protracted subclinical *C. psittaci* infections were found to have a measurable impact on animal health and development (reviewed in [3]). The related human pathogen *C. pneumoniae* is a frequent cause of usually mild respiratory infections and has also been associated with vascular disease. In the case of *C. trachomatis*, different serovars are responsible for ocular, urogenital and respiratory tract infections (reviewed in [4]).

Chlamydiae are obligate intracellular bacteria with a biphasic developmental cycle. Upon contact with host mucosal cells, the infectious but metabolically inactive extracellular form, i.e. the elementary body, induces its uptake into phagosomes. The bacteria block phago-lysosomal fusion and reside as metabolically active reticulate bodies within an intracellular inclusion. The productive cycle ends with the release of a new generation of elementary bodies, i.e. in cell culture after about two days (reviewed in [4]).

In accordance with this intracellular life cycle, specific cellular immunity including a Th_1_-polarized response is essential in host defense against *C. psittaci*. Humoral immunity is, at least in related species such as *C. muridarum* [5]–[7], usually considered to be of minor importance, although specific antibodies can influence the Th_1_ response via Fcγ-receptors and transferred serum from recovered infected mice can enhance functions of CD4^+^ T-cells in genital reinfection [8]–[11].

The complement system (CS) is central to immune effector functions against microorganisms and modulates the intensity of innate and specific immunity (reviewed in [12]). More than 30 complement proteins are found in serum and other body fluids. Receptors for complement components and regulators are expressed on the host-cell surface.

Lipopolysaccharides and other bacterial cell wall components, C reactive protein, necrotic cells, mitochondria, and immune-complexes elicit one of the three major pathways of complement activation. Classical, Alternative, and Mannan-Binding-Lectin Pathway merge at the level of an inactive precursor, i.e. complement factor 3 (C3), which is cleaved upon activation into C3a and C3b. Highly reactive free C3b can covalently bind to surfaces of pathogens. Phagocytes can then detect them via complement receptors (CR) resulting in opsonization and increased uptake. C3b also combines with C3- and C5-convertase. The end point of this cascade is the lytic membrane attack complex (MAC), to which bacteria such as *Neisseria meningitides* are highly susceptible. The cleavage products C5a and C3a promote inflammation and immune modulation. These anaphylatoxins induce signaling by binding to C5a-receptor (C5aR; CD88) [13], [14] and C3a-receptor (C3aR) [15], [16]. C5a is a powerful chemotaxin for granulocytes, but also necessary for the optimal generation of antiviral CD8^+^ T-cell responses [17]. C3aR expression was found in activated T cell clones [18]–[24]. Both anaphylatoxins stimulate granulocytes, monocytes and macrophages, and modulate DC function and Th_1_/Th_2_/Th_17_ polarization. Altogether, CS is highly protective against infection by extracellular pathogens. It can also have an effect on infections with facultative intracellular bacteria [25]–[27]. On the other hand, over-activation of the complement cascade can have deleterious effects [23], [28]–[30].

*In vitro*, it was shown that elementary bodies of *C. trachomatis* can activate the CS [31], [32], while the complement protein properdin can bind to *C. pneumoniae* and accelerate the Alternative Pathway. Moreover, complement reduced chlamydial infectivity in cell culture [33]. *In vivo*, however, it is not clear whether the CS affects the course and outcome of chlamydia infection. The role of complement was not further investigated by others because of ‘discouraging early data’ obtained on *C. muridarium* using decomplementation with cobra venom factor. However, this toxin causes only transient C3-deficiency [6]. Furthermore, it is unknown whether the CS, while acting in extracellular fluids, can significantly influence infections with obligate intracellular bacteria in general. Chlamydiae reside within intracellular inclusions for the larger part of their life cycle. When we started this study on *C. psittaci* we assumed a preferential targeting of the extracellular and metabolically inactive elementary bodies and hypothesized that the CS might provide some protection, or, alternatively, might enhance chlamydial uptake into host cells.

Using complement factor, receptor knockout and deficient mice, the present study demonstrates an important, unexpected and much more complex role for the CS downstream of C3 and upstream of C5 in *C. psittaci* infection of the murine lung.

## Materials and Methods

### Chlamydial Culture

Strain DC15 of *C. psittaci* was isolated from a case of bovine abortion [2]. Its identity was established using 16S rRNA and ompA gene sequencing and recently confirmed by genome sequencing (GenBank accession number CP002806.1). After propagation within BHK-21 cells (kindly provided by B. Sodeik, Hannover, Germany) in PANSERIN 401 serum-free medium (Cytogen, Berlin, Germany) without cycloheximide for 2 days, the infected cells were mechanically disrupted by glass beads. The cell debris was removed by centrifugation, and bacterial aliquots (crude homogenate) were stored in sucrose-phosphate-glutamate solution at −80°C. Infectivity measured as inclusion-forming units (IFU) was determined by titration in HeLa cells as previously described [34]. For mock infection controls, BHK-21 cells were processed identically but without chlamydiae and diluted in PBS at the same ratio as used for inoculated cell culture. PCR confirmed that preparations were mycoplasma-free.

### Experimental *C. psittaci* Infection in Mice

All animal experiments were approved by the Local District Government and carried out in strict adherence to the German regulations and guidelines for the protection of animal life (Permit number: 33.9-42502-04-05/940 and 09/1624). In the *C. psittaci* lung infection model, 10- to 12-week-old male mice of the following strains in the C57BL/6J background were used: C3^−/−^ (B6.129S4-C3tm1Crr/J) [35], C5aR^−/−^ (B6.129S4-C5ar1tm1Cge/J) [36], as well as C57BL/6J wild-type (WT) mice (Charles River WIGA, Sulzfeld, Germany). C5-deficient (C5^def^) Hc0 mice (B10.D2-Hc0 H2d H2-T18c/oSnJ) [37], as well as the corresponding Hc1 WT control mice (B10.D2-Hc1 H2d H2-T18c/nSnJ), both in the B10 background. Unless otherwise stated, intranasal infection was performed using 10^4^ IFU or 200 IFU (low-IFU infection) of *C. psittaci* DC15 in 0.9% NaCl at a final volume of 30 µl per mouse. Mice were anesthetized with isoflurane vapor first and then injected intraperitoneally with 1% Ketamine (Graeub, Albrecht, Aulendorf, Germany) and Midazolam-Actavis (Actavis Group PTC ehf.). Body weight and clinical score of the mice were evaluated on a daily basis. Briefly, mice were assessed for the following parameters: vocalization, piloerection, attitude, locomotion, breathing, curiosity, nasal secretion, grooming and dehydration. Dysfunction in each parameter was rated as one or two points. The body condition of the mice was determined by summating all points, which resulted in a clinical score from untroubled (0 point) to moribund (≥11 points) (in which case the mice were euthanized) [34], [38].

### Serum Transfer Experiment

C57BL/6J mice serving as donors were infected intranasally twice within four weeks with 4×10^4^ IFU of *C. psittaci* DC15 before blood was collected by heart puncture. The sera from several mice were pooled, centrifuged, heat inactivated (56°C, 1 h), and aliquots were stored at −20°C. The resulting hyperimmune serum tested positive in an anti-*C. psittaci* ELISA up to a 1∶5,000 dilution as compared to control serum from non-infected mice, which was negative up to a 1∶25 dilution (data not shown). C3^−/−^ mice as serum recipients were challenged with an intermediate dose of 4,000 IFU per mouse 1 or 7 days before i.v. transfer of 250 µl of 1∶2 NaCl-diluted anti-*C. psittaci* pool or control serum. To determine survival rates, the recipient mice were observed for up to 6 weeks.

### Lung Histopathology

Mice were euthanized on the days indicated, usually on day 4, 9 or 21 after infection and lungs were fixed in 4% buffered formalin (pH 7.2). After dehydration (using a Shandon Hypercenter XP, Thermo Fisher Scientific, Dreieich, Germany), the lungs were embedded in paraffin. Sections (4 µm thick) were deparaffinized with xylene, stained with H&E and then analyzed by light microscopy with all samples blinded. The degree of inflammation was scored according to 1.) the number of inflammatory cells, 2.) the spread and localization of interstitial inflammation (perivascular, peribronchial or patchy), 3.) the area involved (<10% up to >75%) for which criterion scores were assigned from 0 to 2, and 4.) the degree of edema, 5.) exudation and 6.) bleeding for which criterion scores were assigned from 0 to 5, resulting in a maximum possible score of 26 points.

### Cell Profile in Bronchoalveolar Lavage Fluid

The mouse trachea was cannulated and the airways were flushed twice with 1 ml ice-cold 0.9% NaCl. Bronchoalveolar Lavage Fluid (BALF) was centrifuged (10 min; 700×g; 4°C) and the total cell number was determined using trypan blue exclusion dye. 1×105 cells in 500 µl NaCl were used for cytospin (700 rpm, 10 min; ThermoShandon Cytospin 4 Centrifuge, Bolton, UK). Slides were fixed and stained with Romanowski stain variant Diff-Quik staining kit (Medion Diagnostic, Duedingen, Swizzerland) according to the manufacturers’ protocol. 300 cells were counted per slide from random fields by light microscopy to calculate und consideration of the absolute cell number per ml the cell profile in BALF.

### Preparation of Lung Homogenate and Determination of the Bacterial Load

The right lung was removed and homogenized in 1 ml chilled NaCl (0.9%). One hundred µl of lung homogenate mixed with 100 µl of PBS containing 6.86% sucrose, 40 mg/ml gentamicin, 0.002% phenol red and 2% FCS was snap frozen and stored in aliquots at −80°C. Using a similar procedure, the spleen was also removed, homogenized and stored.

For the measurement of bacterial burden by titration, thawed lung (or spleen) homogenate was serially diluted and centrifuged onto HeLa cell monolayers growing on slides. After 24 h in culture, the slides were washed, fixed in methanol and stored at −20°C before staining with chlamydia-specific antibody (Pathfinder Chlamydia culture confirmation system, BioRad, Munich, Germany). The IFU/ml was determined by immunofluorescence microscopy.

Additionally, aliquots of spleen homogenate were DNA extracted using the High Pure PCR Template Preparation Kit (Roche Diagnostics, Mannheim, Germany) and examined by real-time PCR [39] to quantitate *C. psittaci* genome copies.

### Determination of Cytokines and Myeloperoxidase in Lung Homogenate

For the quantification of mouse IFN-γ, TNF-α and IL-2, -4, -6, -10, -12 and -17A in lung tissue homogenate, a Th_1_/Th_2_/Th_17_ Cytometric Bead Array (CBA) kit (BD Biosciences, San Diego, CA) was used according to the manufacturer’s instructions. In brief, the frozen lung homogenate was thawed, mixed with 1 µl protease inhibitor and centrifuged at 5,000×g for 10 min at 4°C to pellet debris. The supernatant was diluted (in duplicate aliquots) at a ratio of 1∶2 in the kit assay diluent. Fifty µl of the sample was mixed with 50 µl of CBA bead mixture in a Highscreen HTS™ BV 96-well filter plate (Millipore, Billerica, MA, USA). After the addition of 25 µl of the kit PE reagent, the plate was incubated for 2 h in the dark. Samples were washed twice with 200 µl of kit wash buffer. Liquid was removed by applying a vacuum, leaving the CBA beads captured in the filter. The beads were washed, resuspended and analyzed on a BD FACSCalibur flow cytometer using FCAP array software (Becton Dickinson, Heidelberg, Germany).

Myeloperoxidase (MPO) concentration was determined using the mouse MPO ELISA Kit (HyCult Biotechnology, Uden, Netherlands). One hundred µl of lung homogenate was mixed with 200 µl MPO-lysis buffer and further processed according to the manufacturer’s instructions. The absorbance at 450 nm was measured from duplicates on a Titertek Multiskan MCC/340 plate reader (Labsystems, Vantaa, Finland).

### ELISAs for the Determination of Plasma Anti-Chlamydia IgM and IgG

Whole blood from heart puncture was mixed with 100 µl of 200 mM EDTA (Sigma, St. Louis, MO, USA) and transferred to a Microtainer® SST vial (BD Biosciences, Heidelberg, Germany). After centrifugation (10,000×g for 10 min) the cell-free supernatant (plasma) was frozen at −80°C.

Nunc 96-well plates (Nunc, Roskilde, Denmark) were coated overnight at 4°C with crude *C. psittaci* homogenate (0.1 µg/100 µl/well). All subsequent incubation steps were performed at 37°C for 1 h. Wells were washed three times with PBS +0.05% Tween. After blocking with BSA and washing, the plates were incubated with 1∶100 diluted plasma samples in duplicate. After washing, HRP-linked antibodies were added for the detection of IgM (clone II/41 BD Biosciences), total IgG (MP Biomedicals, Eschwege, Germany), IgG_2a_ (clone R19-15, BD Biosciences) or IgG_1_ (clone X56, BD Biosciences). Mouse mAb of defined Ig subclasses fixed to the 96-well plate served as a positive control (data not shown). After incubation with substrate buffer, the enzymatic reaction was blocked with H_2_SO_4_. Samples and standards were measured at 450 nm as described above. Aliquots of pooled plasma from infected BL6 WT mice (day 21 post infection (p.i.) were used to obtain a standard curve and to calculate arbitrary units.

### ELISA for Plasma C3a

Mouse C3a (and its more stable, dearginated form C3a_desArg_) as surrogate marker of complement activation was detected by sandwich ELISA using anti C3a neo-epitope-specific capture antibody (clone I87-1162, Becton Dickinson, Heidelberg, Germany) and biotin-conjugated anti-C3a mAb (clone I87-419, Becton Dickinson), followed by streptavidin peroxidase. Arbitrary units of C3a were calculated from a C3a standard curve generated from C57BL/6J EGTA plasma maximally activated by zymosan. Activated Mg^2+^ EGTA plasma from C3^−/−^ mice was used as a negative control to confirm the specificity of the assay (data not shown).

### Sample Size and Statistics

In each set of experiments at each time-point, the analysis was based on n = 9–16 animals for the mice infected with 10^4^ IFU of *C. psittaci* or n = 8 for the animals infected with 200 IFU, and on n = 4–8 for the corresponding mock controls. In the graphs depicting body weight and clinical score, the day 4, 9 and 21 groups were combined for each mouse strain and treatment.

First, a two-way ANOVA was used to compare infected and mock-infected mice. If a significant difference was found for a parameter, more detailed statistical analysis was carried out to evaluate the effects of *C. psittaci* infection in the knockout mouse strains in comparison to the corresponding WT controls. Numeric parameters were analyzed by one-way repeated measures ANOVA; non-parametric parameters such as clinical and histological scores were analyzed using ANOVA on ranks. Post hoc analysis was performed using the Bonferroni method. Survival rates were statistically analyzed using the Log-Rank Mantel-Cox test. Differences of p<0.05 between different strains of infected mice were considered significant and indicated in the graphs by an asterisk (*). For statistical analysis, GraphPad Prism V5 (GraphPad Software Inc., La Jolla, USA) was used.

## Results

### Intranasal Infection with *C. psittaci* DC15 Leads to Complement Activation with Early, Elevated and Sustained C3a Levels in the Plasma

Preliminary experiments had shown that C57BL/6J mice were highly susceptible to the DC15 strain of *C. psittaci*. After intranasal application of 10^4^ IFU, the symptoms of severe pneumonia appeared within one week (data not shown). When the levels of the C3 cleavage product C3a/C3a-desArg were measured in the plasma of these mice and compared to the mock-infected group, they were highly elevated from day 2 on, and were maintained on days 6 and 14 p.i. (Table 1). This proved for the first time that chlamydia infection *in vivo* had rapidly activated the CS and indicated maintained activation at a high level.

**Table 1:** 2way ANOVA for effect of *C. psittaci* DC15 infection: p<0.001.

	C3a (Units)					
	*C. psittaci* DC15 (1E4)			Mock infection		
Day p.i.	Mean	SEM	n	Mean	SEM	n
**2**	596.5	342.0	4	56.8	19.4	4
**6**	535.5	421.9	6	47.6	16.9	4
**14**	856.3	616.9	6	47.0	7.2	4

Further investigation of the consequences of complement activation and the dependency of the clinical course of *C. psittaci* infection on a functioning CS was therefore warranted. For this purpose, *C. psittaci* lung infection was analyzed in knockout mice strains that lacked an individual complement component or receptor.

### In the Absence of C3, Mice are ∼100 Fold More Susceptible to *C. psittaci*

**Figure 1:**
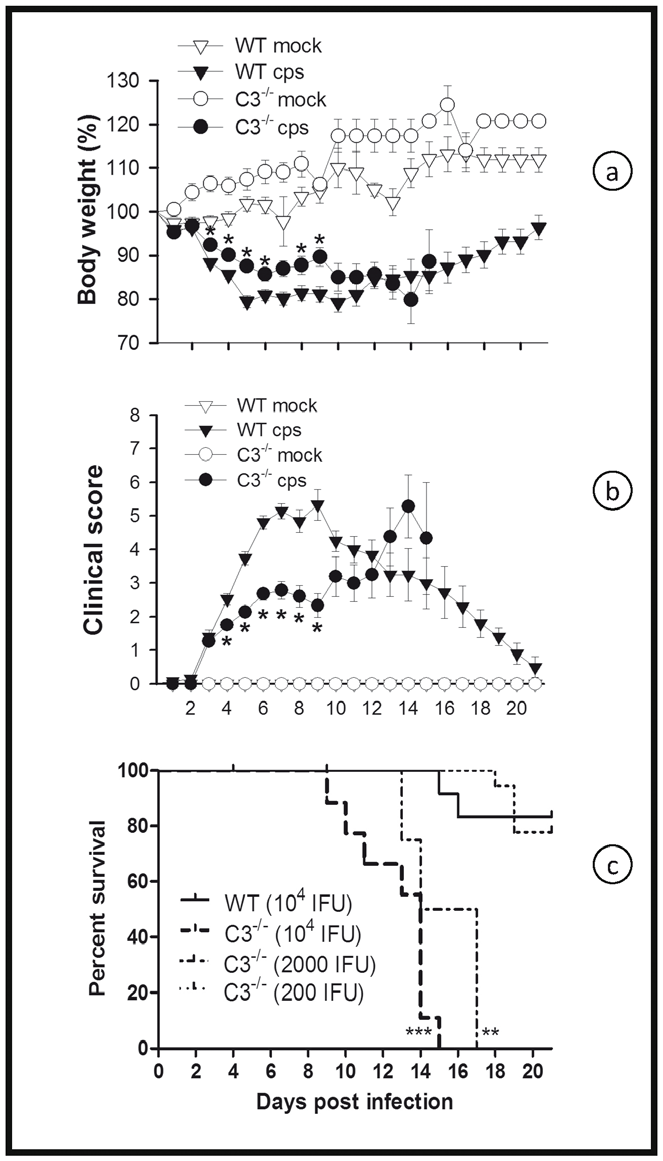
Figure **1. C3 is deleterious during early** ***C. psittaci*** **lung infection, but protective in the late phase.** C3^−/−^ and the corresponding BL/6J wild-type (WT) mice were either intranasally infected with 10^4^ IFU of *C. psittaci* DC15 (*cps*; filled symbols) or mock-infected (*mock*; open symbols) (Fig. 1a and b). In panel c, varying amounts of *C. psitttaci* (10^4^, 2000 or 200 IFU per mouse) were applied as indicated. Body weight (a), clinical score (b) and survival rates (c) were determined for up to 21 days post-infection. Depicted are the means ± SEM. * indicates significant differences (p<0.05) between infected C3^−/−^ as compared to the corresponding infected BL/6J WT mice, and ** or *** p<0.01 and p<0.001, respectively.

C3^−/−^ mice lack a functional CS due to the absence of the common key factor of all three main activation pathways. Along with BL/6J WT mice, the C3^−/−^ mice were intranasally infected with 10^4^ IFU of *C. psittaci* DC15. During the first 9 days p.i., C3^−/−^ mice temporarily lost less body weight and had a slightly but significantly better clinical score compared to WT mice, (Fig. 1a and b). However, intriguingly, between days 9 and 17, all C3^−/−^ mice rapidly deteriorated and died, whereas >80% of the WT mice showed improved clinical scores and body weight and recovered (Fig. 1c).

To investigate the sensitivity of C3^−/−^ mice to *C. psittaci* DC15, a dosis range from 10^4^-200 IFU was tested. Even when infected with the low dose of 200 IFU, approximately 40% of the C3^−/−^ mice died after the second week, indicating that they were almost 100 times more susceptible to *C. psittaci* than mice with a functional complement cascade (Fig. 1c).

### Bacterial Burden, Histological Score, the Granulocyte Marker Myeloperoxidase in the Lung Homogenates of C3^−/−^ Mice, and Differential Cell Content in BALF in High Dose (10^4^ IFU) Infection

**Figure 2:**
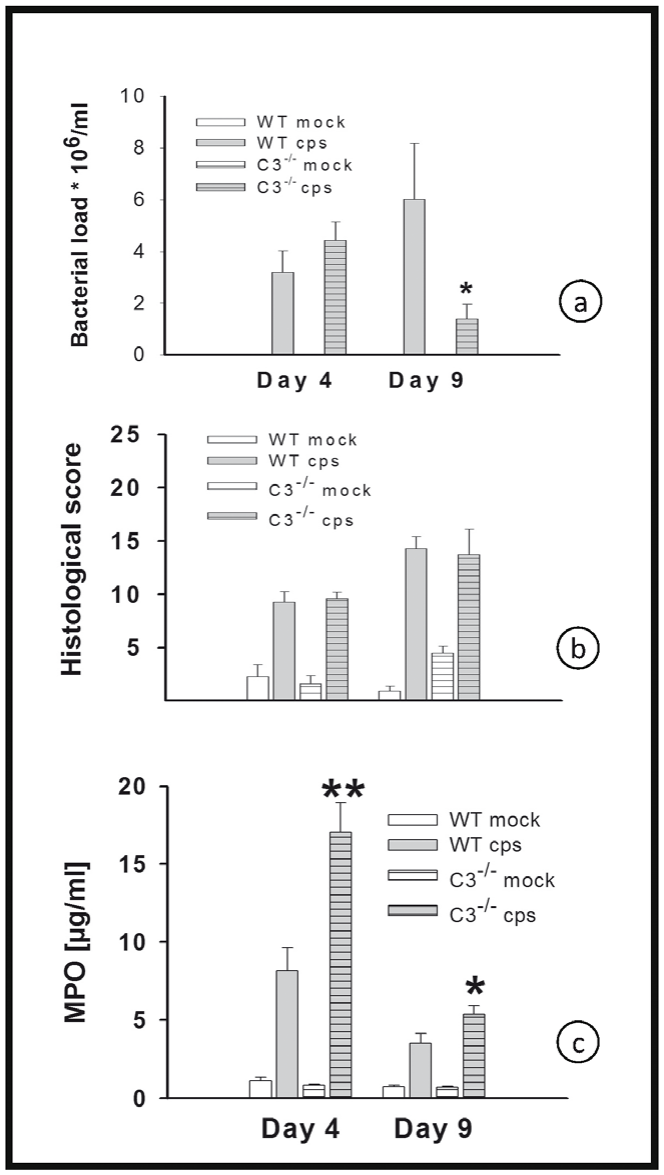
Figure **2. Bacterial load, histological score and MPO of C3^−/−^ and WT mice in high-dose infection.** Mice were infected with 10^4^ IFU of *C. psittaci* DC15 or mock-infected and sacrificed on days 4 or 9 p.i. Bacterial load (panel a), histological score (panel b) and granulocyte marker enzyme MPO (panel c) in lungs of C3^−/−^ and WT mice were determined. The figure shows data obtained from *C. psittaci*-infected mice (cps, gray bars), mock-infected mice (white bars), WT mice (open bars) and C3^−/−^ (horizontally hatched bars). Means ± SEM are depicted. * indicates significant (p<0.05) differences between infected knockout as compared to the corresponding infected BL/6J WT mice.

The bacterial load of C3^−/−^ mice infected with 10^4^ IFU of *C. psittaci* was determined from lung homogenates on days 4 and 9 p.i. It was significantly reduced on day 9 p.i. in C3^−/−^ mice compared with infected WT mice (Fig. 2a). On days 4 and 9, the histological score of the lungs of all analyzed infected mice was significantly elevated compared to the mock controls, but there was no significant difference of infected C3^−/−^ mice compared to infected WT mice (Fig. 2b). MPO was used as a marker enzyme to determine migration of granulocytes into the lung in a more quantitative fashion. Its level was elevated in lung homogenate of infected mice. Moreover, MPO was significantly elevated in the lungs of C3^−/−^ mice as compared to the corresponding WT controls (Fig. 2c). The results of the MPO assay were essentially confirmed for a smaller subset of mice by cell counting of granulocytes in HE-stained lung tissue sections (data not shown).

**Figure 3:**
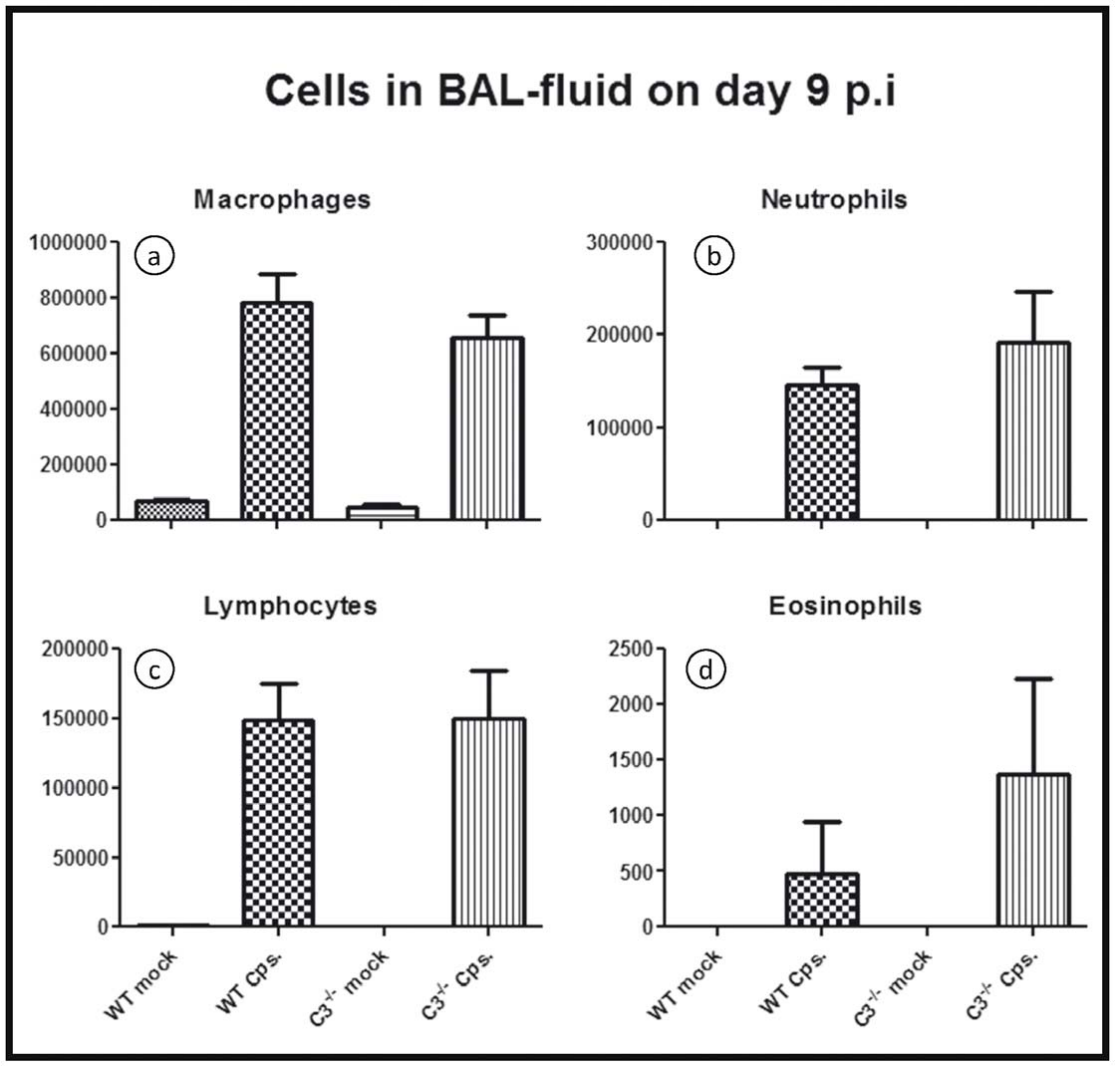
Figure **3. Inflammatory cells in BALF on day 9 p.i. in high-dose infection.** Mice were infected with 10^4^ IFU of *C. psittaci* DC15 or mock-infected and sacrificed on day 9 p.i. The differential cell content (number of cells) was determined in Diff-Quik stained cytospin slides of broncho-alveolar lavage fluid. Means ± SEM are depicted.

To get more information about the inflammatory cells involved at a later stage of *C. psittaci* lung infection, additional mice were infected to obtain BALF on day 9 p.i. for cell staining and counting (Fig. 3). Chlamydial infection caused a drastic increase of macrophages, neutrophils, lymphocytes and eosinophils as compared to mock infected mice. However, there were no significant differences comparing the numbers of the neutrophils, macrophages, or lymphocytes in infected WT and C3^−/−^ mice. In addition there was only a trend for higher numbers of eosinophils in the infected C3 knockout.

### Cytokine Profile in the Lung Homogenate of C3^−/−^ Mice in High Dose Infection

**Figure 4:**
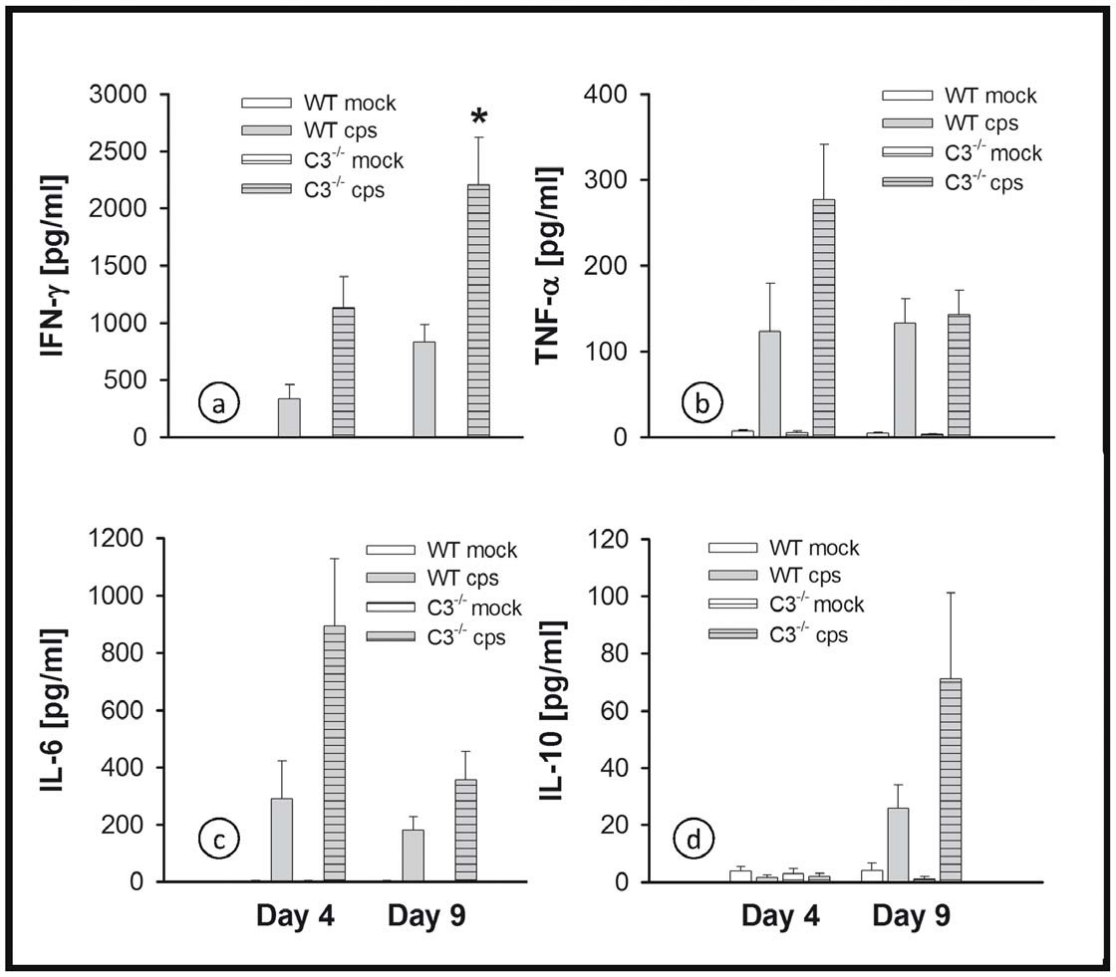
Figure **4. No indication of lack of cytokine response in the absence of a functional complement system.** Mice were infected with 10^4^ IFU of *C. psittaci* DC15 or mock-infected. The cytokines IFN-γ, TNF-α, IL-6 and IL-10 (panel a-d) were determined in the lung homogenate of the animals sacrificed on day 4 or 9. The figure shows data obtained from *C. psittaci*-infected mice (cps, gray bars), mock-infected mice (white bars), WT mice (open bars) and C3^−/−^ (horizontally hatched bars). * indicates significant (p<0.05) differences between infected knockout as compared to the corresponding infected BL/6J WT mice. Means ± SEM are depicted. * indicates significant (p<0.05) differences between infected knockout mice as compared to the corresponding infected BL/6J WT mice.

Of eight cytokines analyzed, a significant difference in levels caused by 10^4^ IFU of *C. psittaci* was found only for IFN-γ, TNF-α, IL-6 and IL-10 (Fig. 4a–d). Therefore, the results for IL-2, IL-4, IL-12 and IL-17α are not shown. In the C3^−/−^ mice the lung tissue concentrations of IFN-γ and TNF-α, two key cytokines in the defense against chlamydia, and of IL-6 and IL-10, were either similar to or even higher than for WT controls. On day 9, IFN-γ had risen to a significantly elevated level in the lungs of C3^−/−^ mice (Fig. 4a).

### Low-dose *C. psittaci* Infection Leads Only in C3^−/−^ Mice to Pneumonia, Additionally its Onset is Delayed

**Figure 5:**
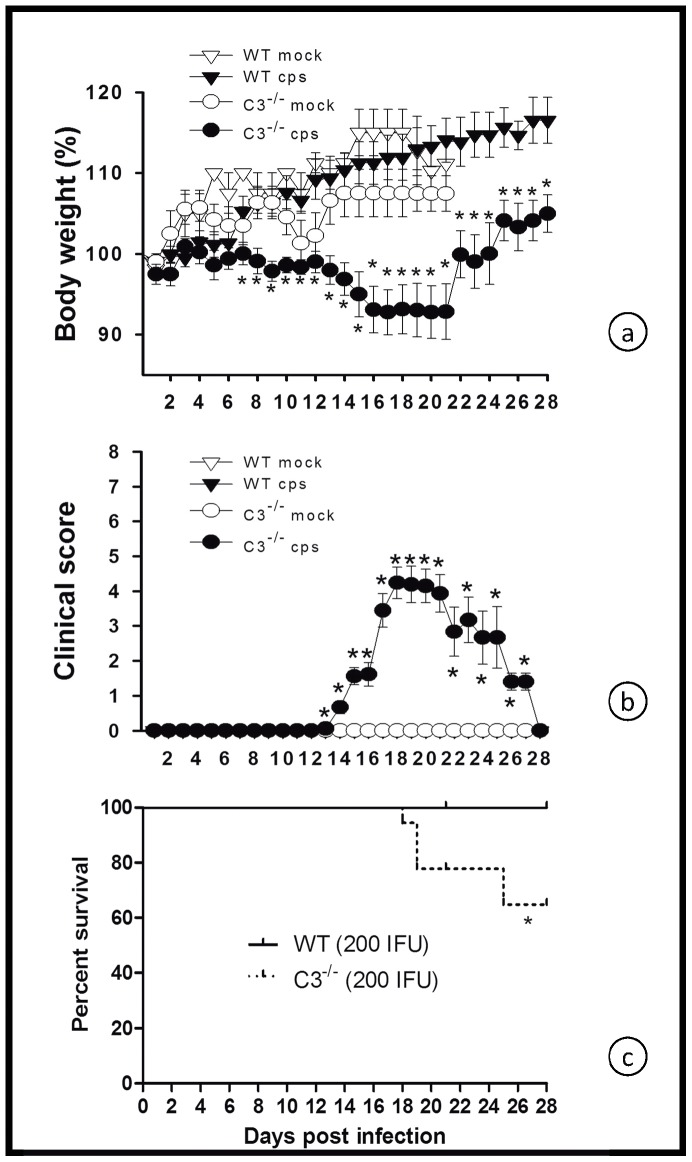
Figure **5. Illness with delayed onset in C3^−/−^ mice in low-dose** ***C. psittaci*** **lung infection.** C3^−/−^ and the corresponding BL/6J WT mice were either infected with 200 IFU of *C. psittaci* DC15 (*cps*; filled symbols) or mock-infected (*mock*; open symbols). Body weight (a) and clinical score (b) were determined daily for up to 28 days. Seventy % of the C3^−/−^ mice survived low-dose infection, as did all WT mice (c). The means ± SEM are depicted. * indicates significant differences (p<0.05) between infected knockout mice as compared to the corresponding infected BL/6J WT mice.

**Figure 6:**
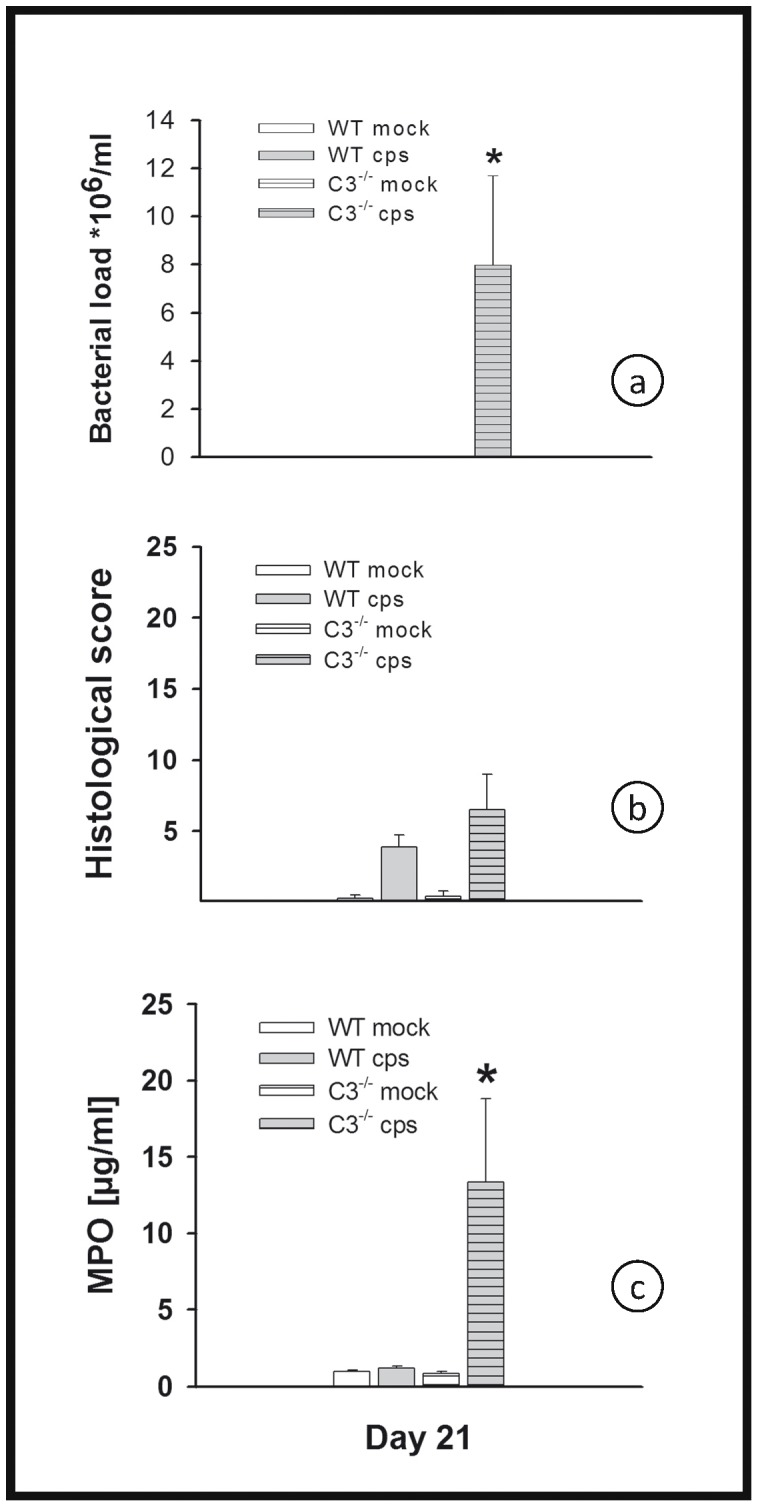
Figure **6. Bacterial load, histological score and MPO in C3^−/−^ and WT mice in low-dose** ***C. psittaci*** **infection.** Mice were infected with 200 IFU of *C. psittaci* DC15 or mock-infected and sacrificed on day 21 p.i. Bacterial load (panel a), histological score (panel b) and MPO (panel c) in C3^−/−^ and WT mice in low-dose *C. psittaci* infection were determined. The figure shows data obtained from *C. psittaci*-infected mice (cps, gray bars), mock-infected mice (white bars), WT mice (open bars) and C3^−/−^ (horizontally hatched bars). Means ± SEM are depicted. * indicates significant (p<0.05) differences between infected knockout as compared to the corresponding infected BL/6J WT mice.

In the 10^4^ IFU infection model, all C3^−/−^ mice died before day 21 (Fig. 1c). Thus, to enable more detailed analysis, the response to infection with a low dose (200 IFU) of *C. psittaci* was also investigated in C3^−/−^ and WT control mice. On day 21, infected WT animals showed no signs of disease (Fig. 5a and b) and had no detectable viable bacteria in the lung (Fig. 6a). Conversely, signs of pneumonia appeared around 14 days p.i. in C3^−/−^ (Fig. 5a and b). Thirty percent of the C3^−/−^ died in low-dose infection between day 17 and 28 ((Fig. 5c), whereas all WT animals survived until day 28 without showing any clinical sign.

Moreover, with an initial dose of 200 IFU per mouse after 3 weeks, about 8×10^6^ IFU of *C. psittaci* were recovered from the lungs of C3^−/−^ mice (Fig. 6a), a similar amount as that observed for WT mice in the high-dose infection model on day 9 (Fig. 2a).

Compared to the mock controls, an elevated histological score in the lung tissue of all mice infected with 200 IFU was apparent on day 21, including in WT mice, which otherwise appeared to be completely healthy. This suggests that even mild chlamydial disease can have long-lasting effects on lung tissue, although the remaining damage was only small in WT mice (Fig. 6b).

MPO was significantly elevated in low-dose infected C3^−/−^ mice on day 21 p.i. compared to WT mice (Fig. 6c).

**Figure 7:**
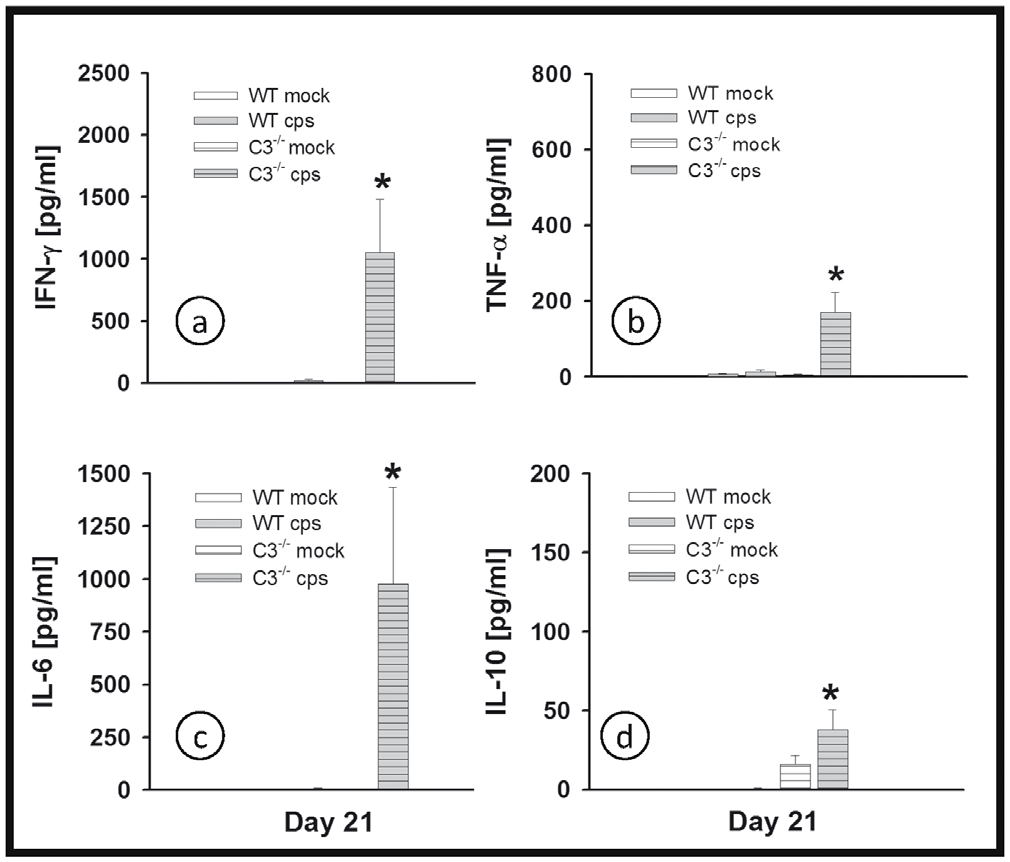
Figure **7. No indication of lack of cytokine response in the absence of complement in low-dose infection.** Mice were infected with 200 IFU of *C. psittaci* DC15 or mock-infected. The cytokines IFN-γ, TNF-α, IL-6 and IL-10 (panel a-d) were determined in the lung homogenate of the animals sacrificed on day 21 p.i. The figure shows data obtained from *C. psittaci*-infected mice (cps, gray bars), mock-infected mice (white bars), WT mice (open bars) and C3^−/−^ (horizontally hatched bars). * indicates significant (p<0.05) differences between infected knockout as compared to the corresponding infected BL/6J WT mice. Means ± SEM are depicted. * indicates significant (p<0.05) differences between infected knockout mice as compared to the corresponding infected BL/6J WT mice.

Corresponding to the delayed but severe pneumonia observed in low-dose infection in C3^−/−^ mice and the increased bacterial load, levels of IFN-γ, TNF-α, IL-6 and IL-10 were significantly elevated in infected animals (Fig. 7, with cytokine levels in a similar range as observed on day 4 or 9 with the high IFU infection (Fig. 4). Again, there was no change detected in IL-2, IL-4, IL-12 or IL-17α due to chlamydial infection in any of the mice (data not shown).

### Dissemination of *C. psittaci* from Lung to Spleen

**Figure 8:**
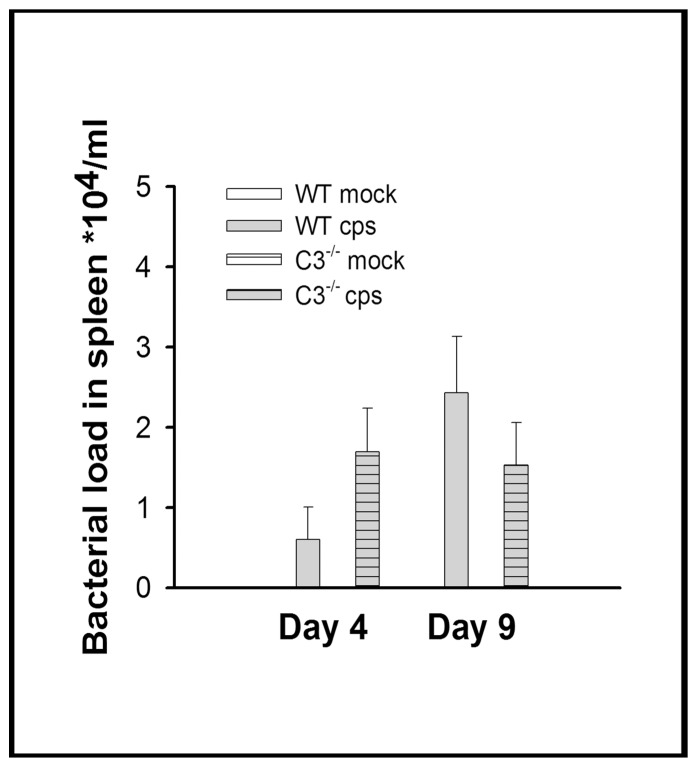
Figure **8. Bacterial load in spleen of infected C3^−/−^ and WT mice.** Mice were infected with 10^4^ IFU of *C. psittaci* DC15 and sacrificed on days 4 or 9 p.i. Bacterial load in spleen of C3^−/−^ (hatched columns) and WT mice (open columns) were determined. A similar pattern as compared to lung homogenate became apparent. However, the differences in bacterial load between infected C3^−/−^ and WT mice were not significant. In spleen of mock-infected mice, no chlamydiae were detected. Means ± SEM are depicted.

To verify whether the infective agent spread from lung to other organ tissue, as is common in human and avian psittacosis, spleen homogenates from day 9 p.i. of the 10^4^ IFU challenge trial were examined by immunofluorescence and quantitative real-time PCR. The bacterial load in chlamydia-positive spleens was in the range of 10^4^–10^5^, i.e. ∼1/100–1/1000 of the amount found in lungs at the same point in time. Viable chlamydiae were detected in most of the spleens analyzed (e.g. 86% in WT), but there was no significant difference between the knockout mouse strain and WT including the bacterial load per spleen and mouse. Nevertheless, the pattern resembled that found in the lung on days 4 and 9 p.i. Mock-infected controls remained negative throughout the trial (Fig. 8). In low-dose infection, viable chlamydiae were still detectable on day 21 in 4/8 (50%) spleens from C3^−/−^ mice, again in the range of 10^5^ IFU, whereas 8/8 WT mice tested negative. Using the more sensitive real-time PCR assay, genomic DNA of *C. psittaci* was detected in all spleen samples of WT and C3^−/−^ mice infected with 10^4^ IFU (data not shown). These observations confirm that *C. psittaci* disseminated in the mice in the course of infection.

### Mice Lacking C3 can Raise a Rather Normal Anti-chlamydia IgM and IgG Response

The unfavorable consequences of C3 deficiency became apparent after approximately ten days in the course of high-dose *C. psittaci* infection (Fig. 1), and even later in low-dose infection (Fig. 5). The delayed onset of pneumonia strongly suggested that impaired adaptive immunity was responsible for the death of or the more severe course in C3^−/−^ mice. Antigen-bound C3b, further processed to C3d, is a B-cell co-activator absent in C3^−/−^ mice that could enhance antibody production in the WT [40].

**Figure 9:**
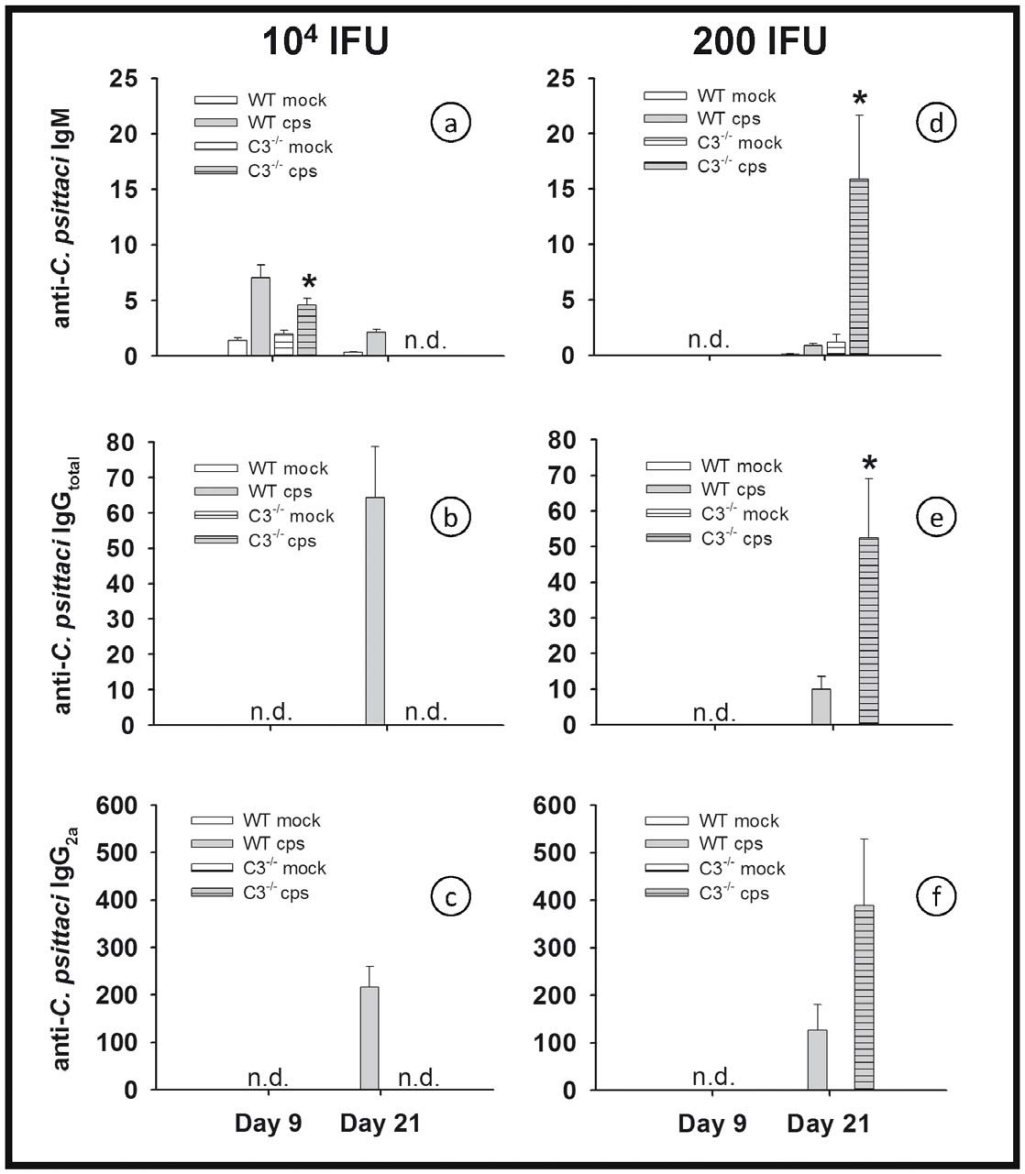
Figure **9. C3^−/−^ mice raise almost normal levels of anti-chlamydial antibodies in low-dose** ***C. psittaci*** **infection.** C3^−/−^ and the corresponding BL/6J WT mice were infected with 10^4^ (panel a–c) or 200 IFU (panel d–f) of *C. psittaci* DC15. Anti-chlamydial IgM (panel a and d), total IgG (panel b and e) and IgG_2a_ (panel c and f) were determined in serum obtained 9 or 21 days p.i. as indicated. All C3^−/−^ mice infected with 10^4^ IFU died before day 21 and were not available for examination (n.d.). Depicted are the means ± SEM. * indicates significant differences (p<0.05) between infected knockout mice as compared to the corresponding infected BL/6J WT mice.

Based on these considerations, the chlamydia-specific humoral response by C3^−/−^ and WT mice was compared. Elevated levels of anti-chlamydia IgM was detected on day 9 in infected WT and C3^−/−^ mice with a significantly lower (or delayed?) increase in the knockouts (Fig. 9a). Total IgG and IgG_2a_ was detected on day 21 p.i. in WT mice infected with 10^4^ IFU of *C. psittaci* (Fig. 9b and c). In contrast, only background levels were present in mock-infected mice and in *C. psittaci*-infected mice on day 4 or 9 (data not shown). Anti-chlamydia IgG_1_ could not be detected even at the lowest dilutions tested on day 21 p.i. in any of the mice (data not shown).

C3^−/−^ mice infected with 10^4^ IFU of *C. psittaci* died before IgG antibody titers could be determined (on day 21 p.i.). Hence, analysis of the humoral immune response was additionally performed on day 21 in low-dose infected mice. In the C3^−/−^ mice, there was a high increase in anti-*C. psittaci* IgM at that time point (Fig. 9d). Anti-chlamydia total IgG and IgG_2a_ was also detectable in the same range or even higher (for IgG_2a_) than observed in serum of WT mice infected with the high dose (10^4^ IFU) infection (Fig. 9e and f). Despite the presence of anti-chlamydial antibodies on day 21, severe clinical signs in C3^−/−^ mice emerged after the second week of infection (Fig. 5), thus suggesting, but not ultimately proving, that the rather normal chlamydia-specific humoral response was non-protective. In order to confirm this suggestion experimentally, a serum transfer step was included after inoculation.

### Protective Effect of Anti-*C. psittaci* Serum IgG Depends on Time Point of Serum Transfer After Inoculation, but is Only Minimal After One Week

**Figure 10:**
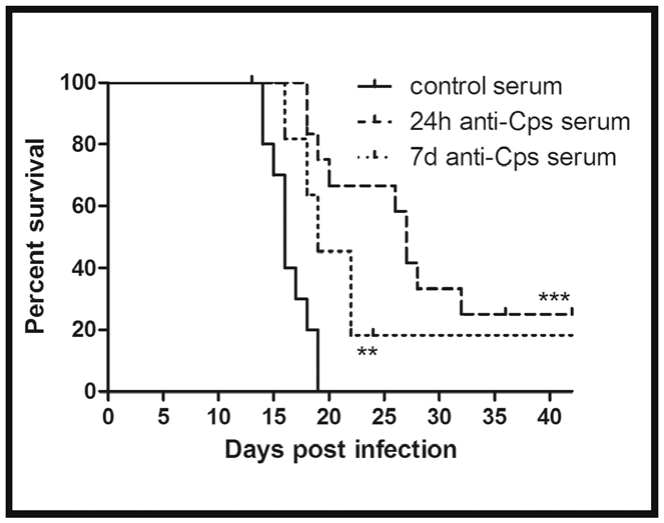
Figure **10. Anti-** ***C. psittaci*** **serum protects C3^−/−^ mice efficiently when transfer is performed within 1 day p.i.** To raise a high-titer immune serum, WT mice were infected with 4×10^4^ IFU *C. psittaci* and re-infected after 28 days. After 56 days, serum of the clinically recovered mice was collected and heat inactivated to eliminate functional donor C3. C3^−/−^ mice were infected with 4,000 IFU of *C. psittaci* and received anti-*C. psittaci* serum either 1 day (dashed line) or 7 days (dotted line) afterwards. Alternatively, heat-inactivated control serum from non-infected mice was transferred to the knockout mice on day 1 p.i. (solid line). Survival rates were determined for up to 42 days post-infection. * indicates significant differences (p<0.05) between mice which received the anti-*C. psittaci* serum as compared to the mice which received control serum, and ** or *** p<0.01 and p<0.001, respectively.

A high-titer anti-*C. psittaci* serum obtained from repetitively infected and recovered WT mice or control serum from non-infected mice, respectively, was transferred i.v. to C3^−/−^ mice. One or seven days before the serum transfer, the recipient mice were challenged intranasally with an intermediate dose (4,000 IFU) of *C. psittaci*. As expected from the dose response experiment (Fig. 1c), all infected C3^−/−^ mice receiving only control serum died in the third week until day 19. In systemic chlamydial disease, relevant antibody production is expected to begin in the second week of infection. Transfer of the anti-chlamydia serum on day 7 p.i., which was intended to simulate the delayed increase in antibodies during bacterial infection, had only little, (but significant) protective effect. In contrast, earlier transfer of anti-*C. psittaci* immune serum on day 1 p.i., which more closely simulates the situation of reinfection after complete recovery, was highly protective and prolonged the mean survival time of the challenged C3^−/−^ mice by more than 1 week (Fig. 10). Consequently, a partial deficiency or a delay in the ‘natural’ IgG increase in the humoral response during lung infection with *C. psittaci* cannot explain the late lethal phenotype of C3^−/−^ mice.

### Mice Lacking Anaphylatoxin C5a Receptor or Complement Factor C5 Respond to *C. psittaci* Lung Infection Almost Like the WT

**Figure 11:**
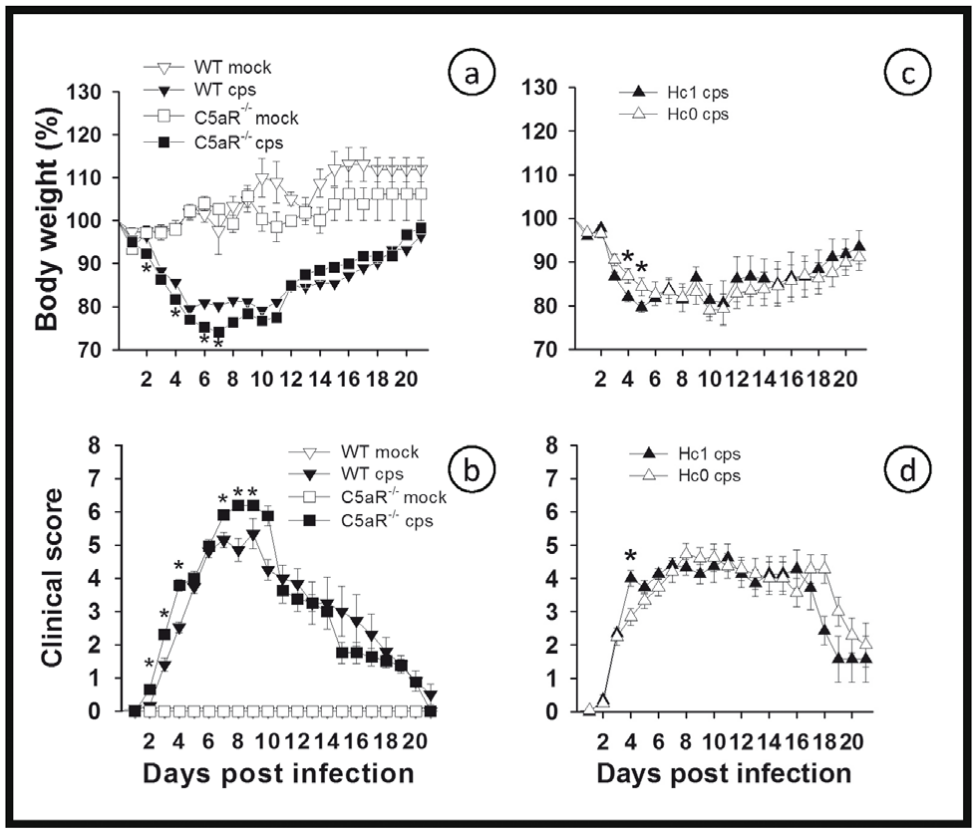
Figure **11. C5aR^−/−^ or C5^def^ mice respond to** ***C. psittaci*** **lung infection almost like the WT.** BL/6J C5aR^−/−^ and the corresponding BL/6J WT mice (a and b) and complement factor C5 deficient mice (Hc0, C5^def^) and the corresponding B10.D2 wild-type (Hc1) mice (c and d), respectively, were intranasally infected with 10^4^ IFU of *C. psittaci* DC15 (*cps*; filled symbols) or mock-infected (*mock*; open symbols). Body weight (a and c), and clinical score (b and d) were determined for up to 21 days post-infection. Depicted are the means ± SEM. * indicates significant differences (p<0.05) between infected knockout or deficient mice as compared to the corresponding infected WT mice.

Central effector functions of the CS downstream of C3 include enhancement of inflammation as well as immune-modulation mediated by anaphylatoxin C5a and its signaling receptor. In contrast to the C3^−/−^ mice, the C5aR^−/−^ mice appeared to have slightly greater weight loss and worse clinical scores compared to WT mice during the early stage of high-dose infection. However, more important, there was no difference in these parameters in the C5aR^−/−^ and WT infected mice after more than 9 days p.i. The C5aR^−/−^ mice neither resembled the phenotype nor the mortality seen in the C3^−/−^ mice at the late stage of infection (Fig. 11a and b). Accordingly, infected C5^def^ mice, unable to generate C5a, were also not different in these two parameters compared to the genetically corresponding Hc1 B10 WT mice (Fig. 11c and d), again with survival rates of >80% and no increase in lethality due to the defect in their CS (data not shown). This result also indicates that MAC (C5b-9) is not required for defense against *C. psittaci* and that the effector function responsible for the late lethal phenotype of C3^−/−^ mice must be downstream of C3, but upstream of C5 within the complement cascade.

## Discussion

During productive infection, host cells are modified and damaged by intracellular chlamydiae. To enable infection of neighboring cells, elementary bodies must be released in the extra­cellular space. Both these facts could contribute to the strong activation of the CS that has been demonstrated here for the first time *in vivo*. In mice infected with *C. psittaci,* C3a levels increased before clinical symptoms were detectable, thus indicating that complement activation could be an early trigger of the inflammatory cascade in chlamydial infection.

Using C3 knockout mice, this study has found that the CS plays an essential and complex role in chlamydial lung disease. Notably, C5aR and MAC have been ruled out as being responsible for the findings obtained with C3^−/−^ mice, in particular for the increased susceptibility and the severe and lethal disease at the late stage of infection. C3a/C3aR and C3b/CRs are the remaining, so far not experimentally excluded known functional members of the CS downstream of C3 and upstream of C5 and, therefore currently the best candidates for future studies.

Moreover, only mice lacking C3 were partially protected early in infection and this early phenotype was also absent in C5^def^ or C5aR^−/−^ mice. Thus, this deleterious early effect of the CS could be due to C3b and its binding to the highly condensed infectious elementary bodies of *C. psittaci*. *Mycobacterium (M.) tuberculosis* activates the CS and binds C3b to its surface [25]. CR3 expressed on macrophages acts as one major phagocytic receptor for the improved uptake of these ‘opsonized’, facultative intracellular bacteria [41]. In contrast to our findings on chlamydiae, the later course of *M. avium* infection is not altered in C3^−/−^ mice during a 5-week observation period [42], which indicates that additional interactions must take place between chlamydiae and the CS. *Chlamydia* (*pneumoniae*) has been demonstrated to use alveolar macrophages and peripheral blood monocytes as ‘Trojan horses’ for covert dissemination [43]. Indeed, as we and others [44] have shown for spleen tissue, *C. psittaci* DC 15 disseminates after lung infection in the host. For this reason, opsonization could be assisting the intracellular bacterium early in infection. The lack of opsonin in C3^−/−^ mice could temporarily counteract the overall negative consequences of a non-functional CS for the host. However, the equal bacterial loads in lung homogenate of C3^−/−^ and WT mice do not support this explanation. The analysis of earlier time points in the mouse model and uptake experiments on purified cells of the two mouse strains in cell culture are needed to address this issue in more detail.

CR3 in cooperation with C3, as well as complement component C1q also augment the uptake and killing of the facultative intracellular bacterium *Listeria (L.) monocytogenes* into pre-activated peritoneal mouse macrophages contributing to improved antigen presentation, and, ultimately, to an enhanced adaptive T-cell response [26], [45]. C3 but not C5a/C5aR promotes the expansion of antigen-specific CD8^+^ and CD4^+^ T-cells in *L. monocytogenes* infection [27]. Similarly, based on decreased antigen presentation and subsequently hampered cellular immunity, C3b/CR3 could also cause the late protective effects in chlamydial infection.

However, it will not be easy to further dissect the role of C3b and CRs using the current model, because there are no knockout mice for each CR available and because some gene deletions do not only affect certain CRs but also other protein complexes with additional functions. As an example, CD11b is not only a subunit of CR3, but also part of an integrin.

The elevated levels of MPO indicate an increase of granulocytes in chlamydia-infected lungs with a peak around day 4. The higher levels, compared to control mice, of MPO (i.e. granulocytes) and certain cytokines in the lungs of C3^−/−^ mice are not that surprising. The observed increase of other chemokines such as IL-6, MCP-1 or KC (data not shown) could be compensating for the lack of C5a/C5aR signaling downstream of C3 in the knockout mice. The anaphylatoxins C5a (and C3a) are not only chemotaxins for granulocytes, but also strong activators of these cells. Thus, even higher levels of MPO/granulocytes in lung tissue of C3^−/−^ mice are not necessarily resulting in higher tissue damage because the activation of these cells might be hampered in the absence of anaphylatoxins. In addition, the decrease of MPO levels in lung homogenate from day 4 to 9, the rather small differences between MPO levels and the similar numbers of granulocytes in BALF of C3^−/−^ and WT mice on day 9, and the almost identical histological scores of infected mice of both strains on that day also argue against a deleterious effect of granulocytes as an explanation of the high lethality in C3^−/−^ mice occurring between day 9 and 17.

In general, the high cytokine levels in the C3^−/−^ mice on day 4 might be partially explained by the slightly higher bacterial load in the lung. However, this cannot be the reason for higher IFN-γ levels on day 9 p.i. when the bacterial load is even significantly lower in the C3^−/−^ mice.

In the time course of the experiments, all mice of the C3^−/−^ strain either died or had to be sacrificed to minimize suffering when the clinical score became higher than 11. Obviously, from the particular day of their death until the originally intended end point of the experiment (on day 4, 9, or 21 p.i.), the animals with the most severe clinical course and thus, most likely, the highest ‘disease activity’ were no longer part of the observed group for daily determination of the clinical score and the body weight. They were also absent in the final assessment of lung tissue for histology and other *in vitro* analyses. As a result, there is a seemingly smaller increase of the clinical score, the loss of body weight and of histological changes and a smaller increase in the concentration of inflammatory mediators in this group. Thus, the effect of the C3 gene knockout at later time-points was most likely even higher than determined by the analysis of the less affected survivors.

The protective effect of the CS in C3^−/−^ mice did not become apparent before the second or third week p.i. This time course indicates that complement activation by chlamydiae is not essential for the immediately acting innate immune defense. Instead, the kinetics suggests a functional link of the CS (and most likely C3a or C3b) to the adaptive and protective immune response in the case of chlamydial infections.

In the low-dose model, C3^−/−^ mice showed signs of severe pneumonia even later then in high dose infection alongside high bacterial load in the lung. Notably, the majority of them recovered and finally survived, in contrast to the 100% lethality in the high dose infection model. Therefore, we hypothesize that complement activation is not an absolute requirement for a specific protective anti-chlamydial response, but that complement might enhance or accelerate it, thereby improving its efficiency.

In the current study, the slightly delayed humoral response (IgG) was ruled out as a putative link between complement and specific immune response. The serum transfer experiment demonstrated that i) pre-existing antibodies can be protective early in infection, i.e. most likely also in *C. psittaci* re-infection, but ii) transferred antibodies alone contribute only minimally to protection later in the course of the ongoing infection with these bacteria. Considering the observed cytokine pattern in the lung homogenate and the unchanged IgG_2a_/IgG_1_ (Th_1_/Th_2_) ratio, there was no indication that a modified Th_1_/Th_2_/Th_17_ polarization took place in C3^−/−^ mice. Nevertheless, future studies including adoptive cell transfer should address in more detail a possible relationship between the CS in chlamydial infection and T-cell (and NK-cell) functions, because the latter are critical factors for the elimination of chlamydiae. This view is supported by data from influenza virus infection, where C3 promoted T-cell priming [46].

As described above, the CS influences the course of infections with facultative intracellular bacteria, such as *Listeria* and *Mycobacteria*. However, most if not all of these interactions, including uptake or invasion by C3b/CR3, are caused by the extracellular, metabolically active form of these bacteria. Therefore, it is straightforward to suggest interaction of these bacteria with the CS, whose components are found in body fluids or are expressed on the surface of host cells. For the ‘late’ phenotype of C3^−/−^ mice observed in infection with obligate intracellular *C. psittaci* (at a time point when adaptive immunity becomes essential in the defense), the situation is different and bound to be more complex.

Preliminary evidence suggests that the observed effect of the CS is not limited to *C. psittaci* infection. Initial experiments in the authors’ laboratory have revealed that C3^−/−^ mice developed an identical phenotype after infection with *C. pneumoniae* strain CWL029 (data not shown). Moreover, a similar functional link might also exist for facultative intracellular bacteria, although this will be more difficult to prove.

In summary, our data suggest that functions of the CS downstream of C3 and upstream of C5 are to some degree harmful in early *C. psittaci* infection. However, more importantly, the results clearly show that in the second and third week p.i., when specific immunity becomes essential for the elimination of *C. psittaci*, complement activation is crucial for a successful defense against these intracellular bacteria. Additional experiments are necessary to elucidate the role of the remaining biologically active cleavage products of C3 (i.e. C3a and C3b) and identify a potential link to the specific cellular immune response against these pathogens. It is possible that the protective effect observed in *C. psittaci* lung infection represents a general mechanism and a specific function of the CS in infections with intracellular bacteria.
